# Effect of intercropping on soil microbial diversity and community network

**DOI:** 10.3389/fmicb.2025.1588559

**Published:** 2025-11-25

**Authors:** Lu Xing Li

**Affiliations:** College of Agronomy Ningxia University, Yinchuan, China

**Keywords:** wheat-soybean intercropping, soil properties, bacterial community, eukaryotic community, co-occurrence networks

## Abstract

**Introduction:**

Understanding the impact of wheat-soybean intercropping on soil microbial communities is crucial for developing sustainable, agricultural practices.

**Methods:**

To investigated how this intercropping system influences soil microbial diversity and network structures, a field experiment was conducted in 2019 using a randomized block design with three treatments: spring wheat monoculture (W), soybean monoculture (S), and 6:2 wheat-soybean intercropping (SW). The soil physical, chemical, and biological properties were analysed, and DNA was sequenced.

**Results and discussion:**

The results showed that the intercropping and sampling location markedly affected bacterial a-diversity, with SW showing a 68.7% higher Shannon index and a 15.0% higher Simpson index than W. Although there were no significant differences in eukaryotic α-diversity and β-diversity between SW and W treatments, unique species distributions were observed. Co-occurrence network analysis revealed that intercropping enhanced the complexity and stability of both the bacterial and eukaryotic communities. Distance-based redundancy analysis (dbRDA) indicated that the soil properties, particularly total phosphorus, available phosphorus, pH, and easily oxidizable carbon, were significantly correlated with the bacterial community composition. While easily oxidizable carbon was the main factor influencing soil eukaryotic community. In conclusion, SW positively regulates soil microbial communities, enhancing bacterial diversity and fostering more stable microbial networks. This study provides a theoretical basis for adopting intercropping to promote agricultural sustainability. Nonetheless, long-term research is needed to explore community shift functions and their long-term impacts on soil health and productivity for sustainable farming.

## 1 Introduction

Soil microorganisms are fundamental to the proper functioning of ecosystems and agricultural systems. They are actively involved in nutrient cycling, the decomposition of organic matter, and the regulation of plant growth. The composition and diversity of soil microbial communities are impacted by a multitude of factors, with cropping systems being a significant one (Ding et al., 2024). Intercropping, which involves cultivating two or more crops simultaneously in the same field, has emerged as a promising agricultural practice. It has been reported to enhance soil fertility, decrease pest and disease occurrence, and contribute to overall agricultural sustainability (Zhu et al., 2019). Wheat (*Triticum aestivum*) and soybean (*Glycine max*) are among the most widely cultivated crops globally, especially in the Yellow River irrigation area in Ningxia, where they play crucial roles in food supply and economic development (Zhu and Morel, 2019). Wheat-soybean intercropping has attracted attention due to its potential advantages in improving soil quality and agricultural productivity (Li et al., 2001; Wang et al., 2022). This practice can modify the physical, chemical, and biological properties of the soil, thereby affecting the structure and diversity of soil microbial communities (Yang et al., 2022).

Further, soil bacteria and eukaryotes are essential for nutrient cycling, organic matter decomposition, and the modulation of plant growth and health through various symbiotic and pathogenic interactions (Aslani et al., 2022; Li et al., 2018). An understanding of the impacts of different cropping systems on these microbial communities is vital for the development of sustainable agricultural practices that enhance soil health and crop yields. In addition, the rhizosphere soil is directly influenced by the plant roots due to the release of root exudates and the presence of plant-microbe interactions (Pini et al., 2017). In contrast, the bulk soil, which is not directly affected by the plant roots, may exhibit a different microbial composition and activity pattern (Pini et al., 2017; Zhang et al., 2023; Zhang C. et al., 2024).

Previous studies have indicated that intercropping systems can increase soil microbial diversity compared to monoculture systems. For example, Zhang L. Q. et al. (2024) demonstrated enhanced bacterial diversity in the rhizosphere of intercropped maize and soybean, in contrast to monocultures. Likewise, Zheng et al. (2024) found greater fungal diversity in the rhizosphere of intercropped wheat and faba bean than in faba bean monoculture. These results emphasize the potential for intercropping to foster a more diverse and resilient soil microbial community, which is beneficial for soil health and crop productivity. However, the mechanism underlying the effects of intercropping and soil sampling location on soil bacteria and eukaryotes remains unclear. This study aimed to elucidate the complex ecological relationships between soil microorganisms, and the environment, and provide a theoretical basis for formulating agricultural adaptation strategies to environmental changes by clarifying the effects of these cropping systems on soil microbial communities. It was hypothesized that (i) wheat-soybean intercropping might change soil microbial taxa and microbial diversity by altering soil physical, chemical, and biological properties; (ii) soil sampling location (bulk and rhizosphere soil) might change soil microbial taxa and microbial diversity by altering soil physical, chemical, and biological properties; (iii) wheat-soybean intercropping system might change soil microbial network complexity and stability by reshaping microbial co-occurrence networks.

## 2 Materials and methods

### 2.1 Site descriptions

The agricultural trial was conducted in March 2019 at Ningxia University’s research farm situated in Xihe village, Wanghong town, within Yongning county of Yinchuan city in the Ningxia Hui Autonomous Region (N 38°14′, E106°14′; 1110 meters above sea level). This region, situated in the northwestern inland area, is characterized by an arid climate typical of the mid-temperate zone. The climatic characteristics include an annual accumulated temperature of 3,300°C, a frost-free growing season spanning 140–160 days, an average of 3,000 h of sunlight per year, a daily temperature fluctuation of 13°C, and an annual rainfall ranging from 180 to 200 mm. The region’s average annual temperature is 8.5°C, with recorded air temperatures ranging from −5.2°C to 36.7°C, respectively. The soil in this area is classified as silty irragic, and according to the USDA soil texture classification, it is categorized as sandy loam. The soil contains 8.5 g kg^–1^ of total organic matter, 0.88 g kg^–1^ of total nitrogen, 0.98 g kg^–1^ of total phosphorus, 18.6 mg kg^–1^ of available phosphorus, and has a pH level of 7.8. Prior to this study, this land was consistently used for wheat cultivation on an annual basis.

### 2.2 Experimental design

A one-factor randomized block design with three replications was employed in this study, with each plot measuring 10 m by 3 m, totalling 30 m^2^. The experimental treatments were organized as follows: 1: spring wheat monoculture, W; 2: spring wheat intercropped with soybean in a 6:2 ratio, SW; 3: soybean monoculture, S. The spring wheat used was *Triticum aestivum* L.var. Ningchun 4, and the soybean was *Glycine max* L.var. Chengde 6. For sowing, 90 kg ha^–1^ of soybean seeds and 338 kg ha^–1^ of wheat seeds were utilized. The wheat was planted with a row spacing of 12 cm, while the soybean rows were spaced 30 cm apart, with individual soybean plants spaced 10 cm apart. The planting density for both wheat and soybean in the intercropped plots matched that of their respective monocultures. Within the intercropping plots, a complete strip consisted of six rows of wheat interspersed with two rows of soybean, with a 20 cm gap maintained between the wheat and soybean strips.

The fertility practices for wheat and soybean cultivation were consistent with local agricultural norms. Before planting, diammonium phosphate containing at least 18% nitrogen and 46% phosphorus pentoxide (applied at a rate of 300 kg per hectare), urea with a nitrogen content of no less than 46% (150 kg per hectare), and potassium magnesium sulfate with a potassium oxide concentration of at least 24% (42 kg per hectare) were uniformly distributed across all test plots. Subsequently, on April 26, 2019, an additional top-dressing of urea, also rich in nitrogen (at least 46%), was manually applied at a rate of 225 kg per hectare. The plots received water on specified dates: April 26, May 16, June 7, and August 29. Throughout the growth period, manual weeding was conducted to manage vegetation competition.

### 2.3 Soil collection

During the wheat flowering stage in 2019 (Philippot et al., 2013; Wang et al., 2017), soil samples were collected from the rhizosphere of both the wheat and soybean plants. A selection of five wheat and soybean plants was gathered from each experimental plot. The roots were carefully shaken to dislodge any surplus soil and then softly brushed to collect the soil closely adhering to the roots (Wang et al., 2017).

Subsequently, these rhizosphere soil samples were bagged, labeled, and chilled on ice before being promptly shipped to a refrigerated storage unit. Each sample underwent sieving with a 2 mm mesh sieve to eliminate plant debris, including roots and rocks, and was then divided into two portions. One portion was stored at 4°C for soil property analysis, while the other was employed for DNA extraction processes.

### 2.4 Measurement of soil properties

#### 2.4.1 Measurement of soil chemical properties

The soil organic carbon (SOC) was analyzed using the potassium dichromate oxidation method (Bao, 2000). Total nitrogen (TN) was determined using the Kjeldahl digestion process (Bao, 2000). Soil pH was measured with a pH meter (Bao, 2000). The total phosphorus (TP) and available phosphorus (AP) concentrations were measured using the molybdenum blue color reaction method, after digestion with a sulfuric acid and perchloric acid blend and extraction with 0.5 M sodium bicarbonate, respectively (Bao, 2000). Soil solution conductivity (Cs) was assessed with a conductivity meter (Bao, 2000). The soil water-soluble organic carbon (WSOC) content was measured using the multiple solid–water ratio method (Bao, 2000). Easily oxidizable organic carbon (EOC) was measured using the potassium dichromate oxidation method (Bao, 2000). The sulfate sulfur (SN) content was measured using a continuous-flow analyser after extraction with 2 M potassium chloride solution (Bao, 2000).

#### 2.4.2 Measurement of soil biological properties

Microbial phosphorus (Pm) was quantified after chloroform fumigation and subsequent extraction with 0.5 M sodium bicarbonate solution at pH 8.5 (Lin, 2004). The soil urea activity was determined using the indophenol colorimetric technique (Lin, 2004). The soil sucrose activity was evaluated with the 3,5-dinitrosalicylic acid color reaction method (Guan, 1986). The catalase activity was measured using the colorimetric method (Trasar-Cepeda et al., 1999). The soil phosphatase activity was measured using the colorimetric method (Guan, 1986).

### 2.5 DNA extraction and sequencing analysis

Soil DNA was isolated from 0.25 g samples of freshly collected soil using a TianGen Soil DNA kit, according to the protocol provided by the manufacturer. The extracted genomic DNA was then assessed for both quantity and quality using a NanoDrop ND-1000 spectrophotometer (Thermo Fisher Scientific, United States) and agarose gel electrophoresis.

For the polymerase chain reaction (PCR) targeting soil bacteria, the forward primer (ACTCCTACGGGAGGCAGCA) and reverse primer (GGACTACHVGGGTWTCTAAT) were utilized to target the V3-V4 hypervariable regions of the bacterial 16S rRNA gene (Claesson et al., 2009). The amplification of the 18S rRNA gene’s V4 variable region in soil protists was achieved using the forward primer CCAGCASCYGCGGTAATTCC and reverse primer ACTTTCGTTCTTGATYRA (Stoeck et al., 2010). The PCR mixture, totalling 25 μL, consisted of 5 × reaction buffer (5 μL), 5 × GC buffer (5 μL), dNTPs at a concentration of 2.5 mM (2 μL), forward primer at 10 μM (1 μL), reverse primer at 10 μM (1 μL), DNA template (2 μL), nuclease-free water (8.75 μL), and Q5 DNA polymerase (0.25 μL). The thermal cycling conditions were as follows: an initial denaturation at 98°C for 2 min, then 25 cycles of denaturation at 98°C for 15 s, annealing at 55°C for 30 s, extension at 72°C for 30 s, and a final extension step at 72°C for 5 min. Agencourt AMPure Beads from Beckman Coulter, United States, and a PicoGreen dsDNA quantification kit from Invitrogen, United States, were employed for purification and quantification of the PCR products, respectively. After individual quantification, equal volumes of the PCR products were pooled and subjected to paired-end 2 × 300 bp sequencing on the Illumina MiSeq platform, utilizing a MiSeq Reagent Kit v3 from Beijing Medical Technology.

### 2.6 Statistical analysis

SPSS software version 17.0 was used to perform a two-factor analysis of variance (ANOVA) to assess the effects of different intercropping configurations on the soil chemical characteristics, enzymatic activities, and microbial phosphorus (Pm). One factor was the sampling location: rhizosphere or bulk soil, and the other factor was the intercropping system: W, S, or SW. Sequencing data analysis was carried out utilizing QIIME2 (Bolyen et al., 2019) and R software, version 4.4.1. The reads were trimmed and filtered to remove low-quality sequences.

For the 16S amplicon data, amplicon sequence variant (ASV) denoising was implemented with DADA2 in QIIME2-2024.5, resulting in an ASV feature table and representative sequences of ASVs. The representative sequences were taxonomically annotated using the SILVA-138.2 database. For the 18S amplicon data, ASV denoising was performed using DADA2 in QIIME2-2024.5, generating an ASV feature table and representative sequences of ASVs. Taxonomic annotation of the representative sequences was carried out using the PR2 database. Within QIIME2, the ASV table was employed to assess various alpha diversity indices, including the Chao1 estimate, Shannon’s diversity index, and Simpson’s index. The R (v4.4.1) package was utilized to create a plot, and the “anova” command was used to compare the α diversity of soil bacteria and soil eukaryotes. The microbial community matrix was calculated with the vegan package using R (Version 4.4.1). Dimensionality reduction analyses were performed using the cmdscale and metaMDS functions, respectively, to obtain coordinate axes. Visualization plots were generated with the ggplot2 package. Permutational multivariate analysis of variance (PERMANOVA) was conducted via the adonis2 function in the vegan package, yielding the F statistic, R^2^ value, and *p*-value.

Linear discriminant analysis (LDA) Effect Size (LEfSe) was conducted to show features at the phylum, class, order, family, and genus levels, to explain differences among different soil sampling locations and intercropping treatments. LEfSe employs a non-parametric factorial Kruskal-Wallis (KW) sum-rank test to show features (phylum, class, order, family, and genus) with significant differential abundance, and then LDA is used to evaluate the effect size of the different features. Features were considered significantly different at a *p* < 0.05 level and an effect size threshold of 2 (on a log10 scale).

Co-occurrence network analysis was conducted to compare the changes in the soil bacteria and eukaryotes among the different intercropping systems, with a correlation coefficient (r) > 0.6 and a false discovery rate-corrected *p* < 0.05 indicating a significant difference. This analysis was performed using the R package (v.4.4.1). Gephi (v.0.10.1) was applied to visualize the co-occurrence networks and to calculate the topology. Network complexity parameter and network stability indices such as complexity, robustness and vulnerability were calculated according to Zhang C. et al. (2024).

Further, distance-based redundancy analysis (dbRDA) was performed using R software to explore the relationships between soil physicochemical properties and soil bacterial and eukaryotic taxa. All the raw sequencing data of the microorganisms were submitted to the NCBI Sequence Read Archive, under ID PRJNA1212860 for soil bacteria and ID PRJNA1213242 for soil eukaryotes.

## 3 Results

### 3.1 Soil properties

Soils under the SW treatment had a 20.6% higher TP content compared to the S treatment (Table 1). There was no difference in TP between the RSW and RS treatments. Intercropping and soil sampling location had no significant effects on TN, SOC, and pH. The intercropping treatment tended to have a higher AP content than the monocropping treatments. Rhizosphere soil also typically had a higher AP content than bulk soil. Intercropped soil generally had lower Cs than monocropping treatments. Rhizosphere soil had higher Cs than bulk soil. Intercropped soil had lower WSOC than monocropped soil. Rhizosphere soil had a higher WSOC content than bulk soil. Intercropped soil had a lower EOC than monocropped soil. Rhizosphere soil had a lower EOC than bulk soil. Intercropped soil had a lower SN than monocropped soil. Rhizosphere soil had a higher SN than bulk soil.

**TABLE 1:** Soil chemical properties under different intercropping systems.

Treatments	TP (g kg^–1^)	TN (g kg^–1^)	AP (mg kg^–1^)	pH	SOC (g kg^–1^)	Cs (us cm^–1^)	WSOC (g kg^–1^)	EOC (mg g^–1^)	SN (mg kg^–1^)
W	1.59 ± 0.13a	1.16 ± 0.08a	44.0 ± 3.8a	8.4 ± 0.75a	9.15 ± 0.67a	27.8 ± 1.2d	0.36 ± 0.03c	2.93 ± 0.24a	27.0 ± 2.4ab
SW	1.58 ± 0.15a	1.20 ± 0.08a	41.1 ± 4.1ab	8.3 ± 0.74a	8.85 ± 0.64a	28.4 ± 2.3d	0.34 ± 0.03c	1.17 ± 0.1c	16.6 ± 1.4c
S	1.31 ± 0.09b	1.12 ± 0.08a	29.9 ± 2.7c	8.2 ± 0.73a	8.87 ± 0.67a	43.6 ± 3.5c	0.54 ± 0.05b	0.99 ± 0.09c	27.9 ± 2.3a
RW	1.31 ± 0.12b	1.23 ± 0.2a	31.2 ± 3.2bc	8.2 ± 0.1a	8.87 ± 0.83a	69.4 ± 6.1a	0.99 ± 0.08a	1.47 ± 0.12b	24.2 ± 2.4b
RSW	1.50 ± 0.12ab	1.15 ± 0.02a	36.8 ± 3.7b	8.0 ± 0.04a	9.37 ± 0.99a	45.1 ± 3.8bc	0.49 ± 0.04b	1.38 ± 0.11b	28.2 ± 1.8a
RS	1.33 ± 0.11b	1.14 ± 0.12a	35.4 ± 3.6bcd	8.1 ± 0.03a	8.52 ± 0.9a	49.0 ± 4.8b	0.50 ± 0.04b	1.41 ± 0.11b	25.4 ± 2.5ab
***F*-value**									
Block	0.14ns	4.16ns	0.8ns	1.63ns	2.48ns	6.3*	2.13ns	4.67*	2.5ns
Sampling location (L)	0.33ns	0.07ns	5.1ns	0.54ns	0.01ns	246.5**	148.09**	30.07**	5.1*
Bulk soil	1.49a	1.16a	38.3a	8.3a	8.96a	33.3b	0.41b	1.69a	23.8b
Rhizosphere	1.38a	1.17a	34.5b	8.1a	8.92a	54.5a	0.66a	1.42b	25.9a
Intercropping systems (T)	4.29*	0.83ns	5.08*	0.14ns	0.56ns	28.8**	56.53**	161.76**	7.89*
W	1.45ab	1.20a	37.6a	8.3a	9.01a	48.6a	0.68a	2.20a	25.6a
SW	1.54a	1.18a	39.0a	8.1a	9.11a	36.8b	0.41c	1.27b	22.4b
S	1.32b	1.13a	32.7b	8.2a	8.7a	46.3a	0.52b	1.20b	26.6a
L × T	2.15ns	0.63ns	9.65**	0.06ns	0.68ns	62.3**	95.28**	138.39**	27.0**

The values represent the mean ± SD. Different lowercase letters in the same column show significant difference among intercropping treatments (*p* < 0.05). “*” and “**” represent significance at 5 and 1% levels, respectively. W, bulk soil in wheat monoculture; SW, wheat intercropped with soybean; S, soybean monoculture; RW, rhizosphere soil in wheat monoculture; RSW, rhizosphere soil in wheat intercropped with soybean; RS, rhizosphere soil in soybean monoculture; TP, The total phosphorus (g kg^–1^); TN, The total nitrogen content (g kg^–1^); AP, soil available phosphorus (mg kg^–1^); SOC, the soil organic carbon content (g kg^–1^); Cs, Soil electrical conductivity (us cm^–1^); WSOC, soil water-soluble organic carbon content (g kg^–1^); EOC, soil easily oxidizable organic matter (mg g^–1^); SN, nitrate nitrogen (mg kg^–1^).

There were significant differences in urease activity among the different treatments. For instance, urease activity was the highest in the RSW treatment, reaching 90.0 mg kg^–1^ h^–1^, while it was the lowest in the S treatment, at 48.6 mg kg^–1^ h^–1^. The urease activity in the rhizosphere soil (80.7 mg kg^–1^ h^–1^) was higher than that in the bulk soil (55.2 mg kg^–1^ h^–1^). The sucrose activity varied among the different intercropping treatments (Table 2). The RSW treatment had the highest sucrose activity, at 2.10 mg g^–1^ h^–1^, while the SW treatment had the lowest, at 0.93 mg g^–1^ h^–1^. The sucrose activity in the rhizosphere soil (1.71 mg g^–1^ h^–1^) was higher than that in the bulk soil (0.99 mg g^–1^ h^–1^). There were significant differences in microbial phosphorus among the different treatments. The W treatment had the highest microbial phosphorus value, at 19.74 mg kg^–1^, while the RS treatment had the lowest, at 1.15 mg kg^–1^. The microbial phosphorus value in the bulk soil was 8.54 mg kg^–1^, and in the rhizosphere soil, it was 1.70 mg kg^–1^. Phosphatase activity differed among the different treatments (Table 2). The RS treatment had the highest phosphatase activity, at 107.2 mg kg^–1^ h^–1^, while the SW treatment had the lowest, at 56.8 mg kg^–1^ h^–1^. The phosphatase activity in the rhizosphere soil, 92.1 mg kg^–1^ h^–1^, was higher than that in the bulk soil, 65.4 mg kg^–1^ h^–1^. The catalase activity varied among the different treatments. The S treatment had the highest catalase activity, at 3.8 g kg^–1^ 20 min^–1^, while the RS treatment had the lowest, at 2.8 g kg^–1^ 20 min^–1^. The catalase activity in the rhizosphere soil was 3.2 g kg^–1^ 20 min^–1^, and in the bulk soil, it was 3.5 g kg^–1^ 20 min^–1^.

**TABLE 2:** Soil microbial properties under different intercropping systems.

Treatments	Urease activity (mg kg^–1^ h^–1^)	Sucrose activity (mg g^–1^ h^–1^)	Microbral phosphorus (mg kg^–1^)	Phosphatase (mg kg^–1^ h^–1^)	Catalase activity (g kg^–1^ 20 min^–1^)
W	62.8 ± 5.9c	1.02 ± 0.09cd	19.74 ± 0.16a	68.7 ± 6.7c	3.3 ± 0.28b
SW	54.2 ± 4.3d	0.93 ± 0.08d	2.30 ± 0.2c	56.8 ± 5.6d	3.5 ± 0.3ab
S	48.6 ± 4.2d	1.02 ± 0.08cd	3.57 ± 0.31b	70.7 ± 6.9c	3.8 ± 0.35a
RW	79.5 ± 7.9b	1.87 ± 0.17b	1.87 ± 0.18cd	90.7 ± 8.1b	3.5 ± 0.29ab
RSW	90.0 ± 8.4a	2.10 ± 0.16a	2.09 ± 0.2cd	78.4 ± 6.4c	3.3 ± 0.27b
RS	72.6 ± 6.2b	1.16 ± 0.1c	1.15 ± 0.11d	107.2 ± 8.7a	2.8 ± 0.23c
***F*-value**					
Block	8.32**	7.94**	4.8*	3.22*	6.15*
Sampling location (L)	160.86**	353.62**	149.7**	85.99**	11.88**
Bulk soil	55.2b	0.99b	8.54a	65.4b	3.5a
Rhizosphere	80.7a	1.71a	1.70b	92.1a	3.2b
Intercropping systems (T)	13.54**	47.48**	12.01**	18.43**	0.81ns
W	71.2a	1.44a	10.8a	79.7b	3.4a
SW	72.1a	1.52a	2.20b	67.6c	3.4a
S	60.6b	1.09b	2.37b	89.0a	3.3a
L × T	7.58**	63.83**	102.74**	2.9ns	11.57**

Different lowercase letters in the same column show significant difference among intercropping treatments (*p* < 0.05). “*” and “**” represent significance at 5 and 1% levels, respectively. W, bulk soil in wheat monoculture; SW, wheat intercropped with soybean; S, soybean monoculture; RW, rhizosphere soil in wheat monoculture; RSW, rhizosphere soil in wheat intercropped with soybean; RS, rhizosphere soil in soybean monoculture.

### 3.2 Soil bacterial community

**FIGURE 1:**
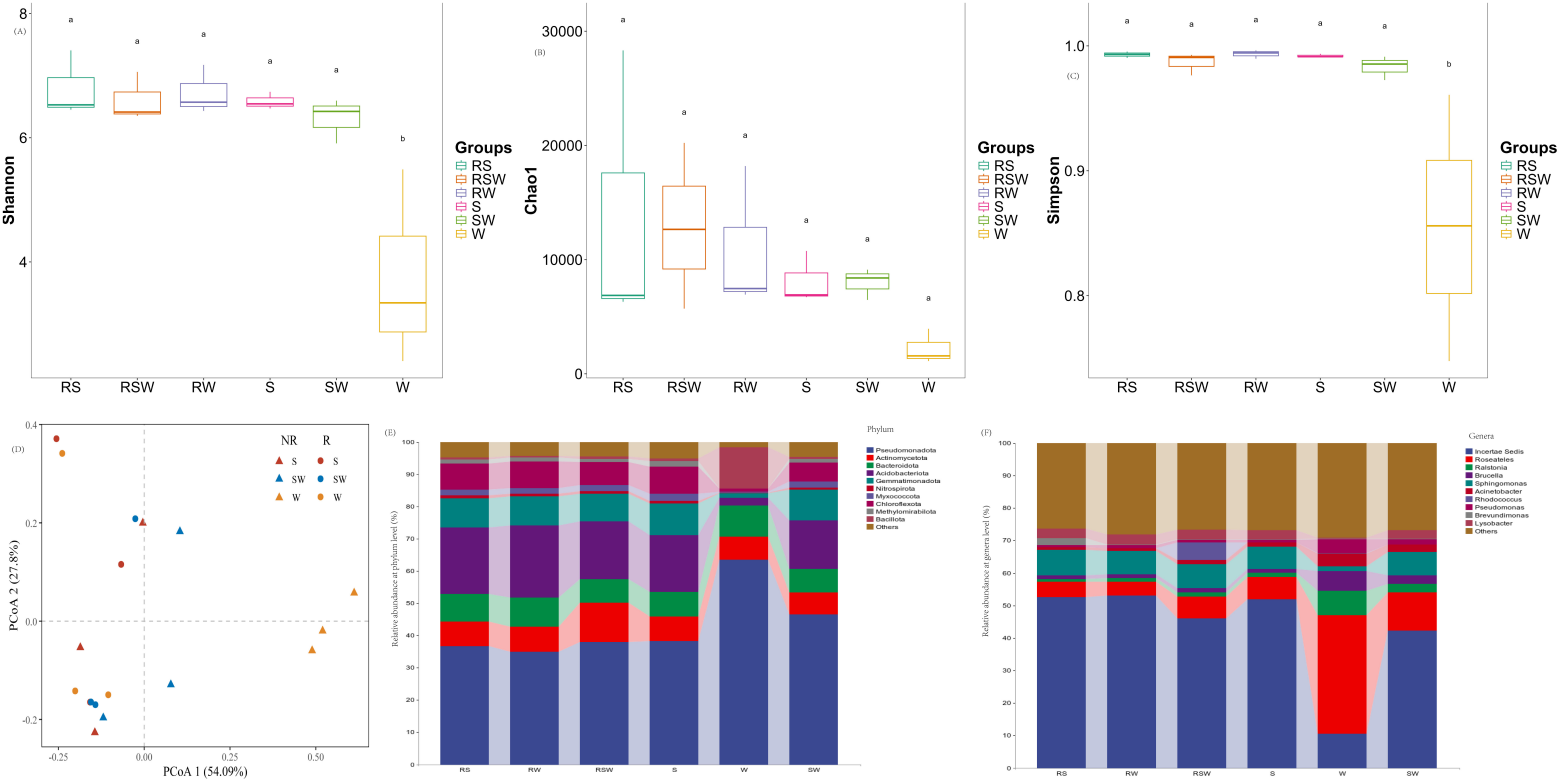
Effect of sampling location and intercropping systems on α-diversity of soil bacterial (ANOVA followed by LSD test; *p* < 0.05). **(A)** Represented Shannon diversity index, **(B)** represented Chao 1, **(C)** represented simpson index, **(D)** represented unconstrained PCoA (PCo1 and PCo2) with Bray–Curtis distance showing the soil bacterial of the RS, RW, RSW, S, W, and SW samples. **(E)** Represented relative abundance of soil bacterial at phylum level, **(F)** represented relative abundance of soil bacterial at genus level.

A total of 55,605 ASVs were obtained from the 18 soil samples. The α-diversity of soil bacterial showed that the Shannon index, and Simpson of soil bacterial communities were impacted by the different treatments (Figures 1A–C; Supplementary Table 1). The lowest Shannon index, and Simpson were recorded in W treatment, meanwhile SW significant increased Shannon diversity index and Simpson by 68.7 and 15.0% as compared to W, respectively. While no difference (*P* > 0.5) in Shannon index, and Simpson was found among RS, RW, RSW, S and SW treatments. Also no difference in Chao1 was recorded among different treatments. For β-diversity, the PCoA showed that soil bacterial communities were significantly different among different treatments (*R*^2^ = 0.19, *F* = 3.77, *p* = 0.013) (Figure 1D; Supplementary Table 2).

**FIGURE 2:**
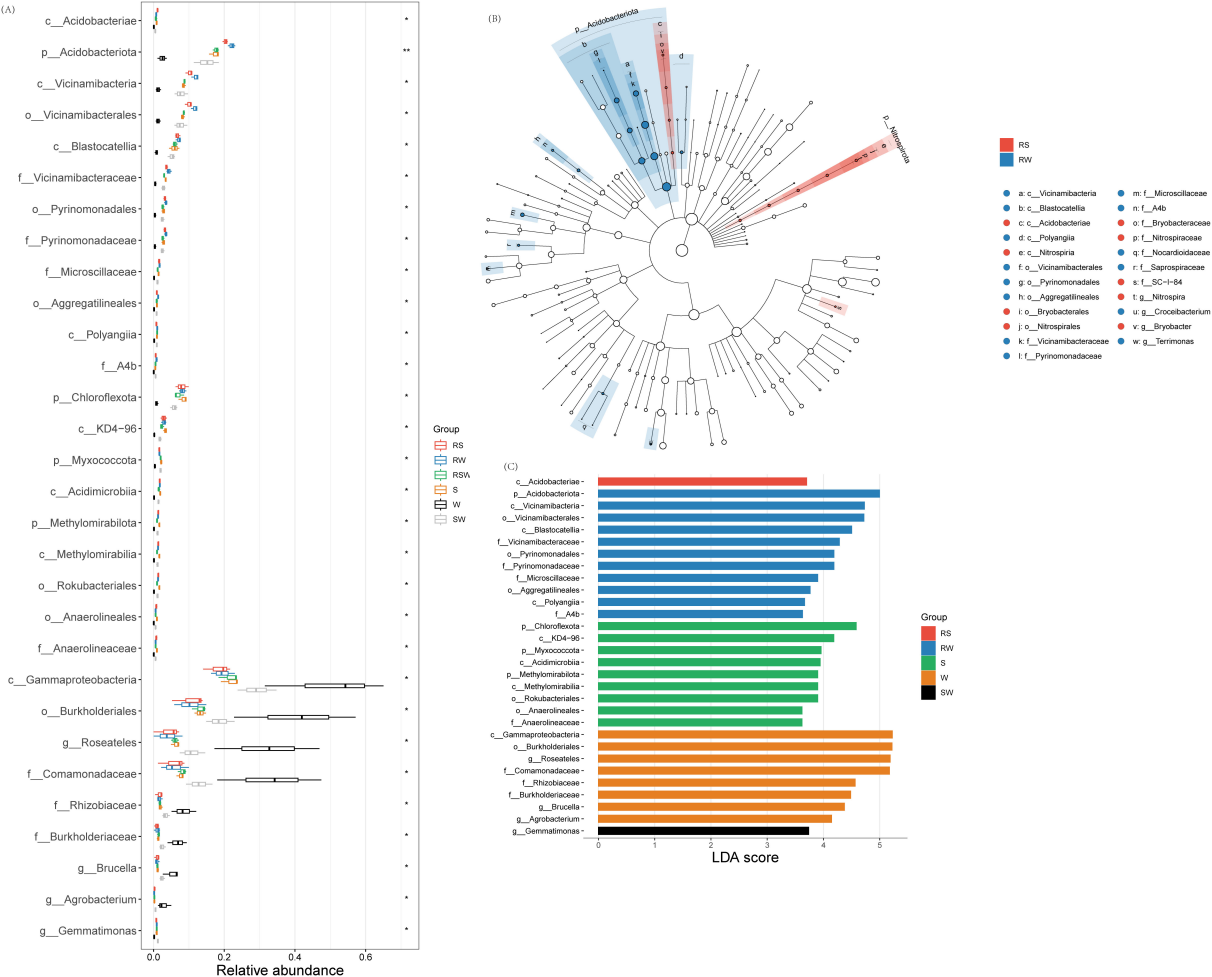
Effect of different intercropping systems on relative abundance **(A)** and Lefse **(B,C)** of soil eukaryotic. Features are ranked by the LDA scores, and blue/green/red bars represented the features enriched in different soil sampling and intercropping plots (*P* < 0.05).

At the phylum level, the top 10 dominant taxa were Pseudomonadota (32.2–82.1%), Actinomycetota (1.5–21.2%), Bacteroidota (4.0–12.6%), Acidobacteriota (1.1–23.2%), Gemmatimonadota (0.6–11.7%), Nitrospirota (0.1–1.0%), Myxococcota (0.1–2.3%), Chloroflexota (0.5–10.0%), Methylomirabilota (0.1–1.9%), and Bacillota (0.2–37.0%) (Figure 1E). At the genus level, the top 10 was Pelomonas (0.02%∼48.07%), Sphingomonas (0.91%∼9.09%), RB41 (0.07%∼3.99%), Lysobacter (0.07%∼4.35%), Gemmatimonas (0.08%∼1.95%), Ochrobactrum (0.25%∼6.88%), MND1 (0.08%∼1.93%), Ralstonia (0.01%∼9.51%), Acinetobacter (0.07%∼7.33%) and Pseudomonas (0.08%∼5.25%) (Figure 1F). The LEfSe results showed that the different genera of soil bacteria between the bulk and rhizosphere soil were Sphingomonas, Ralstonia, and Roseateles (Figure 2; Supplementary Figure 1). The phylum with the highest LDA score in RW was Acidobacteriota (Figure 2; Supplementary Table 3) (LDA = 5.0, *p* < 0.01), Chloroflexota (LDA = 4.58, *p* < 0.05), Myxococcota (LDA = 3.96, *p* < 0.05), Methylomirabilota (LDA = 3.90, *p* < 0.05) were enriched in the S system (Figure 2C; Supplementary Table 3). At the genus level, Gemmatimonas (LDA = 3.74, *p* < 0.05) was enriched in the SW treatment, while Roseateles (LDA = 5.19, *p* < 0.05), Brucella (LDA = 4.37, *p* < 0.05), and Agrobacterium (LDA = 4.14, *p* < 0.05) were enriched in the W treatment (Figure 2C; Supplementary Table 3).

### 3.3 Soil eukaryote community

**FIGURE 3:**
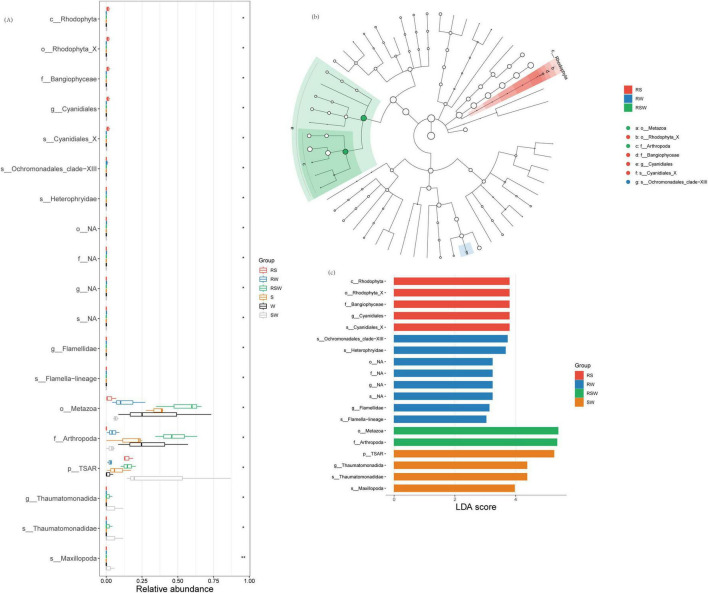
Effect of different intercropping systems on relative abundance **(A)** and Lefse **(B,C)** of soil eukaryotic. Features are ranked by the LDA scores, and blue/green/red bars represented the features enriched in different soil sampling and intercropping plots (*P* < 0.05).

A total of 1,799 ASVs were obtained from the 18 samples. No differences in α diversity indexes of soil eukaryotic between W and SW treatments (Supplementary Figure 2 and Supplementary Table 1). Although the overall composition of the soil eukaryotic communities was similar between the bulk soil and rhizosphere soil (Supplementary Table 2) (*F* = 0.79, *R*^2^ = 0.047, *p* = 0.75), and among the different cropping systems (Supplementary Table 2) (*F* = 1.05, *R*^2^ = 0.12, *p* = 0.38), the LEfSe results showed that the different species of soil eukaryotes between the bulk and rhizosphere soil were Cercomonadidae, Hartmannellidae, Leptophryidae, Allapsidae, Chilodonellidae, Limnofilidae, and Thaumatomonadidae (Figure 3A and Supplementary Figure 3). The different genera were Vampyrellida, Cercomonadida, Cyrtophoria_4, Limnofilida, and Thaumatomonadida (Figure 3A and Supplementary Figure 3). The different families were Endomyxa and Phyllopharyngea. Besides, the different species of soil eukaryotes among the different planting systems were Chrysophyceae, Ochromonadales, Sordariomycetes, Maxillopoda, Filamoebidae, Phalansteriidae, and Insecta, and the different genera were Chrysophyceae, and Pezizomycotina (Figures 3A, B and Supplementary Figure 3). The different families were Coccidiomorpha and Filosa-Sarcomonadea. There was one different class (Rhizaria) and one different phylum (TSAR) (Figure 3A). The different orders were Apicomplexa and Cercozoa. The genus with the highest LDA score in SW was *Thaumatomonadida* (LDA = 4.38; *p* < 0.05) (Figure 3C; Supplementary Table 4).

### 3.4 Soil bacterial network complexity and stability

**FIGURE 4:**
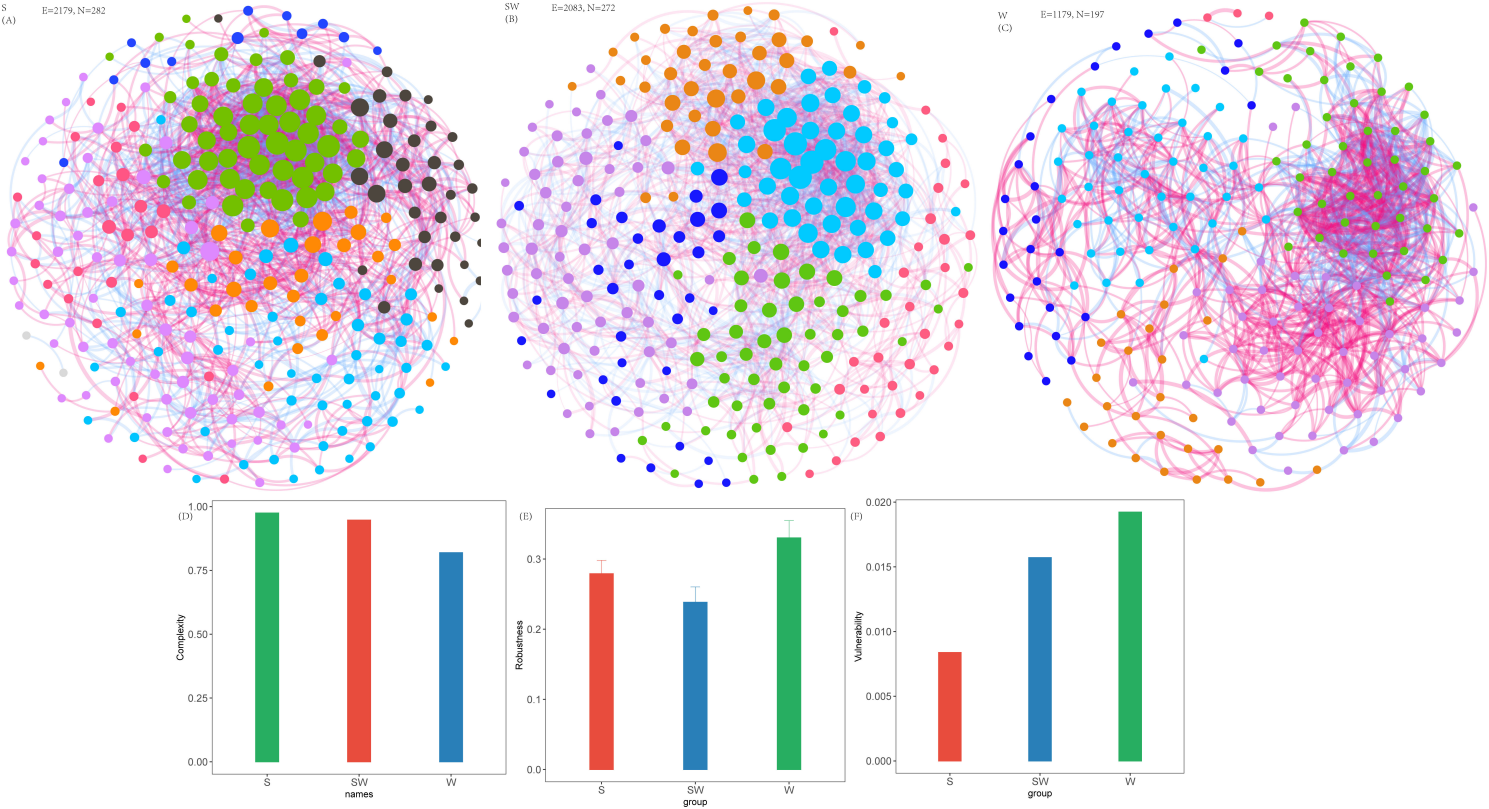
Soil bacterial network, network complexity index and stability under different intercropping systems. **(A)** Soil eukaryotic network under soybean monoculture (S). **(B)** Soil eukaryotic network under wheat intercropped with soybean (SW). **(C)** Soil eukaryotic network under wheat monoculture (W). **(D)** Network complexity index. **(E)** Robustness calculated as 50% of the taxa randomly removed from each network. **(F)** Network vulnerability. Where nodes indicate different ASVs, and edges between the nodes indicate significant interactions. Node colors represent different phylum, and the node size represents the number of degrees connected with the node. Link colors represent the various interactions between soil microbial species, including positive (red) and negative (blue) interactions.

Co-occurrence networks of soil bacteria were constructed (Figure 4; Supplementary Table 5). For the soil bacterial community, the S system exhibited the highest comprehensive complexity (complexity = 0.98) (Figure 4A; Supplementary Table 5), with statistically significant maximum values among the three cropping systems in terms of the number of vertices (282), number of edges (2179), number of positive edges (1227), and clustering coefficient (0.51) (Figure 4A; Supplementary Table 5). The SW system ranked second in complexity (complexity = 0.95) (Figure 4B; Supplementary Table 5); with numbers of vertices (272) and edges (2083) were relatively close to those of the S system, but it possessed a greater number of negative edges (1019). The complexity was 0.82 in W system, and the number of vertices, number of edges, average degree, and clustering coefficient were 197, 1179, 11.97, and 0.49, respectively. With respect to network robustness, when the vertex removal proportion reaches 0.5 (i.e., 50% of vertices lost), the remaining connectivity of the SW system was 0.238, that of the S system was 0.279, and that of the W system was 0.330 (Figure 4E; Supplementary Table 6). The vulnerability assessment results follow the order: S (0.0084) < SW (0.0157) < W (0.0192) (Figure 4F; Supplementary Table 7).

### 3.5 Co-occurrence networks of soil eukaryotic

**FIGURE 5:**
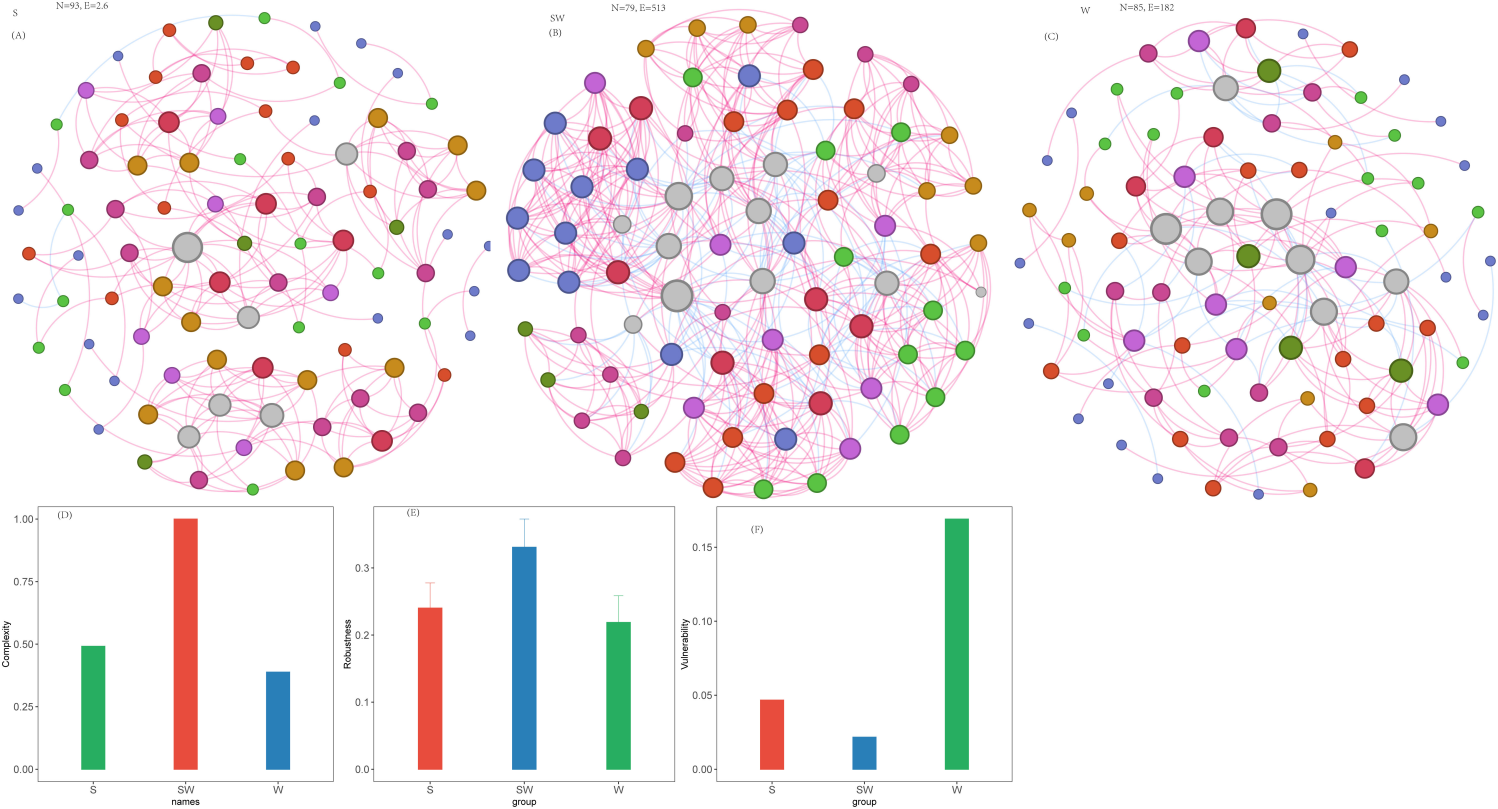
Soil eukaryotic network, network complexity index and stability under different intercropping systems. **(A)** Soil eukaryotic network under soybean monoculture (S). **(B)** Soil eukaryotic network under wheat intercropped with soybean (SW). **(C)** Soil eukaryotic network under wheat monoculture (W). **(D)** Network complexity index. **(E)** Robustness calculated as 50% of the taxa randomly removed from each network. **(F)** Network vulnerability. Where nodes indicate different ASVs, and edges between the nodes indicate significant interactions. Node colors represent different phylum, and the node size represents the number of degrees connected with the node. Link colors represent the various interactions between soil microbial species, including positive (red) and negative (blue) interactions.

Co-occurrence networks of soil eukaryotes were constructed. For the soil eukaryotic community, the SW system exhibited the highest complexity (complexity = 1.0), with the highest values among the three cropping systems in terms of the number of edges (513), average degree (12.99), and clustering coefficient (0.65) (Figure 5; Supplementary Table 5). In contrast, the S system had moderate complexity (complexity = 0.49). Although it had the largest number of vertices (93), it had a relatively low number of edges (206) and average degree (4.43), accompanied by extremely few negative edges (only 3). The W system, meanwhile, exhibited the lowest complexity (complexity = 0.38), with the lowest minimum values in links (182), number of positive edges (140), average degree (4.28), clustering coefficient (0.37) (Figure 5; Supplementary Table 5).

With regard to network robustness, the results indicated that when the vertex removal proportion was 0.5 (i.e., 50% of vertices lost), the remaining connectivity of the SW system was 0.331, that of the S system was 0.240, and that of the W system was only 0.219 (Figure 5E; Supplementary Table 5). Furthermore, when the removal proportion reached 0.9, the remaining connectivity of the SW system (0.036) was higher than that of the S system (0.011) and that of the W system (0.012). The vulnerability assessment results were as follows: SW (0.0216) < S (0.0467) < W (0.1690). Quantitatively, the vulnerability of the eukaryotic network in the W system was 7.8 times that of the SW system, rendering it extremely prone to structural collapse under external disturbances. In contrast, the SW system maintained the lowest vulnerability, which, combined with its high complexity and robust connectivity retention, confirms that its eukaryotic network stability was the most optimal among the three cropping systems.

### 3.6 Relationship between soil properties and microbial communities

**FIGURE 6:**
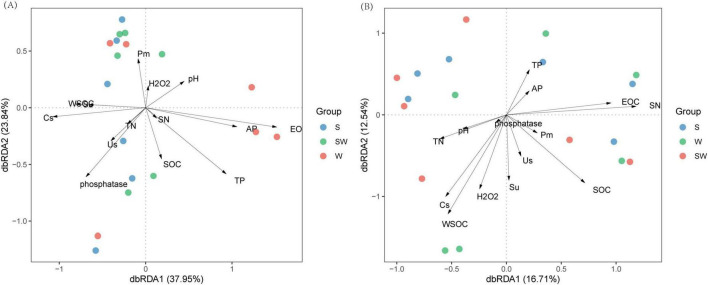
Distance-based redundancy analysis (dbRDA) of soil bacterial **(A)**, soil eukaryotic **(B)** at amplicon sequence variants (ASVs) level and soil properties. W, wheat monoculture; SW, wheat intercropped with soybean; S, soybean monoculture; TP, The total phosphorus (g kg^– 1^); TN, The total nitrogen content (g kg^– 1^); AP, soil available phosphorus (mg kg^– 1^); SOC, the soil organic carbon content (g kg^– 1^); Cs, Soil electrical conductivity (us cm^– 1^); WSOC, soil water-soluble organic carbon content (g kg^– 1^); EOC, soil easily oxidizable organic matter (mg g^– 1^); SN, nitrate nitrogen (mg kg^– 1^); Us, The soil urease activity (mg kg^–1^ h^– 1^); Su, the soil sucrose activity (mg g^–1^ h^– 1^); Pm, the soil microbral phosphorus (mg kg^– 1^); H_2_O_2_, the catalase activity (g kg^– 1^ 20 min^– 1^).

dbRDA was performed to examine the relationships between soil bacterial and eukaryotic community compositions at the ASV level and soil properties. The results showed that for the bacterial community composition, the total explanatory power of the first and second axes was 61.79%, indicating that soil properties provided a good explanation of bacterial community composition (Figure 6A). Total phosphorus (TP), available phosphorus (AP), pH, and easily oxidizable carbon (EOC) significantly affected the bacterial community composition (*P* < 0.05), with explanatory degrees of 45.6, 21.2, 27.1, and 25.0%, respectively. In contrast, the activity of sucrase (Su) and water soluble organic carbon (WSOC) were significantly negatively correlated with the bacterial community composition (*P* < 0.01), with explanatory degrees of 32.4 and 38.9%, respectively (Figure 6A; Supplementary Table 8).

For the soil eukaryotic community composition, the contribution rates of RDA1 and RDA2 were 16.71 and 12.54%, respectively, with a combined explanatory rate of 29.25%. Easily oxidizable carbon (EOC) significantly affected the eukaryotic community composition (*P* = 0.048) with an explanatory degree of 51.9% (Figure 6B; Supplementary Table 9). This indicated that the variations of the soil bacterial and eukaryotic community composition might be affected by the changes of soil chemical and biological properties induced by the cropping systems.

## 4 Discussion

### 4.1 Soil bacterial community diversity

This study revealed that the α-diversity of soil bacterial communities, including the Chao1, Shannon, and Simpson indices, was significantly influenced by both the sampling location and cropping system. The lowest Shannon and Simpson were observed in the W treatment, suggesting that wheat monoculture systems may lead to a reduction in soil bacterial diversity. This is potentially due to the increased complexity and resource availability in intercropped systems. This finding is consistent with recent studies that have shown that intercropping systems can enhance soil microbial diversity and stability. However, Peralta et al. (2018) showed that corn-soybean-wheat + 2 cover crops decreased soil bacterial diversity by 4% as compared to monoculture corn, this might be due to different crop, soil texture, and climate.

At the phylum level, Pseudomonadota was the dominant taxon. This is in agreement with other studies that have identified Pseudomonadota as a major component of bacterial communities in agricultural soils (Liang et al., 2024). The increase in Pseudomonadota in bulk soil compared to rhizosphere soil, irrespective of the cropping system, is contrary to the study of Zhang and Zhang (2024), which found that compared to bulk soil, rhizosphere soil had an increased relative abundance of Pseudomonadota by 31.65%. This difference could be related to the cropping systems and climate conditions. The dominance genera of Gemmatimonas in the intercropped system may indicate their role in nutrient cycling and soil health (Ma et al., 2024). Similarly, other studies also suggested a shift in community composition that could enhance ecosystem functions such as nutrient cycling and disease suppression (Ma et al., 2024; Yang et al., 2024).

The dbRDA revealed that TP, AP, pH, and EOC are key factors for bacterial communities, highlighting the importance of phosphorus availability and soil acidity in shaping bacterial composition. This is similar to the findings of Keet et al. (2021), who emphasized the correlation between phosphatase activities and soil bacterial microbial communities. Besides, a structural equation model indicated that the variations of the soil bacterial community composition was mainly related to an increase in soil acid cations in subtropical forest ecosystem (Li et al., 2019). Similarly, other study also reported that phosphatase is crucial for releasing inorganic phosphorus from organic compounds, and its activity level can directly impact the availability of this essential nutrient for microbials (Liang et al., 2020).

### 4.2 Soil eukaryote community diversity

Soil eukaryotes play crucial roles in soil nutrient cycling, energy flow, and maintaining ecosystem stability (Bardgett and van der Putten, 2014), In this study, a total of 1,799 ASVs were obtained from 18 samples, providing a solid foundation for analyzing the structure and diversity of soil eukaryotic communities. Despite the lack of significant differences in the α- and β-diversity of soil eukaryotes at first glance, the LEfSe analysis revealed that among the differential taxa between rhizosphere and bulk soils, taxa such as Cercomonadidae, Hartmannellidae, and Thaumatomonadida showed significant differences. These taxa are often related to soil nutrient cycling and microbial interactions (Bonkowski, 2004). Besides, other study showed that root exudates, a rich cocktail of organic compounds, sugars, and signaling molecules secreted by plant roots, create a unique microenvironment in the rhizosphere (Pini et al., 2017). This specialized niche selectively favors or disfavours certain eukaryotic organisms, leading to the observed differences at the species, genus, family, order, and class levels between bulk and rhizosphere soil. Intercropping alters the soil resource availability, root architecture, and below-ground interactions compared to monoculture (Latati et al., 2016). The differential abundance of eukaryotic taxa could be due to competition for resources, allelopathic effects, or symbiotic relationships established between different crops and soil organisms. The higher abundance of Arthropoda in RSW implies that specific intercropping setups can favor certain phylogenetic groups, and this might have cascading effects on soil functions like decomposition and nutrient mobilization (Yang et al., 2024). Notably, the genus of Thaumatomonadida had the highest LDA score in the SW treatment. This indicates that Thaumatomonadida is a key indicator taxon for the SW intercropping system. Thaumatomonadida are known to be involved in soil microbial food webs, and their enrichment in SW may imply specific interactions between crops and soil microbial communities, which could potentially enhance soil ecological functions (Bonkowski, 2004).

The dbRDA revealed that EOC was the main factor influencing soil eukaryotic community composition. Similarly, Zhao et al. (2018) reported that the abundance of soil eukaryote communities was impacted by the soil’s physical and chemical properties, such as O-elements, water content, and soil organic matter. Higher TN can support a more diverse community by fuelling biosynthesis, while WSOC provides an easily accessible carbon source for heterotrophic eukaryotes (Zhao et al., 2018). Overall, these relationships highlight the need for a holistic understanding of soil ecosystems, considering both chemical and biological aspects, to predict and manage soil health and productivity.

### 4.3 Soil microbial network characteristics

This study systematically evaluated the effects of different cropping systems on the structure and stability of microbial networks by constructing co-occurrence networks of soil bacteria and eukaryotes. The results showed that in the bacterial network, the soybean monocropping (S) system had the largest number of vertices, edges, and the highest clustering coefficient, indicating a denser network structure and more frequent interspecific interactions among species. This is similar to the study by Qiao et al. (2024), who found that leguminous crops can promote the enrichment of specific bacterial taxa through rhizosphere deposits, enhance positive interactions among microorganisms. Although the robustness of the S was better than that of the W, it had the lowest vulnerability, suggesting that its network is more resistant to random vertex removal. This is consistent with Hernandez et al. (2021) who suggested that highly complex networks may be more prone to reconstruction when facing disturbances.

In contrast, in the eukaryotic network, the wheat-soybean intercropping (SW) system exhibited the highest complexity, robustness, and the lowest vulnerability, higher number of negative edges, which is similar to the study of Morriën et al. (2017) who showed that negative interactions help maintain the dynamic balance of the network. Such distinctive structural traits of the soil biotic network are likely to foster functional differentiation at a fine scale within the community. The vulnerability result showed that even after massive vertex loss, eukaryotes in the SW system still maintained relatively high network connectivity, exhibiting significantly stronger resistance to random interference compared to the S and W systems. Thus, the wheat-soybean intercropping system might play a key role in maintaining soil ecosystem functions and disturbance resistance by promoting the structural optimization and stability improvement of the eukaryotic network. Although the soybean monocropping system showed high complexity in the bacterial network, its eukaryotic network had high vulnerability, thus, the response of different microbial taxa should be comprehensively considered when evaluating the ecological effects of cropping patterns. However, future studies on the mechanistic links between microbial network structure and ecosystem functions is needed to provide a theoretical basis for sustainable agricultural management.

## 5 Conclusion

The study systematically investigated the influence of a wheat-soybean intercropping system on soil chemical and microbial properties and soil bacterial and eukaryotic communities. The results demonstrated that SW significantly increased the soil TP content by 19.8% and altered AP, electrical conductivity, WSOC, EOC, and nitrate nitrogen as compared with soybean monoculture. Relative to W, SW significantly enhanced the bacterial Shannon and Simpson indices by 72.6 and 14.0%, respectively. β-diversity analysis further confirmed significant differences in the bacterial community structure among the treatments. At the phylum level, Pseudomonadota was the dominant taxon, showing higher relative abundance in bulk soil. Intercropping also significantly influenced the relative abundances of multiple phyla, including Acidobacteriota and Gemmatimonadota. LDA revealed distinct bacterial and eukaryotic taxa across the different treatments and sampling locations. Co-occurrence network analysis demonstrated that SW resulted in soil bacterial and eukaryotic networks with higher complexity and greater stability compared to W. The dbRDA identified soil TP, AP, pH, and EOC as key factors driving the bacterial community structure, while EOC was significantly correlated with the eukaryotic community composition. Therefore, promoting wheat-soybean intercropping holds significant practical value for achieving sustainable agricultural development. However, future long-term field experiments are recommended to further elucidate the functional consequences of microbial community succession in intercropping systems and their long-term ecological benefits.

## Data Availability

All data has been released to the NCBI with PRJNA1212860 for soil bacterial and PRJNA1213242 for soil eukaryotic.
