# Supplementary material for: Effect of intercropping on soil microbial diversity and community network
Source: Front Microbiol. 2025 Nov 25;16:1588559. doi: 10.3389/fmicb.2025.1588559 (PMC12685828; doi:10.3389/fmicb.2025.1588559)
Supplement: Supplementary file 1 [file Supplementary_file_1.docx]

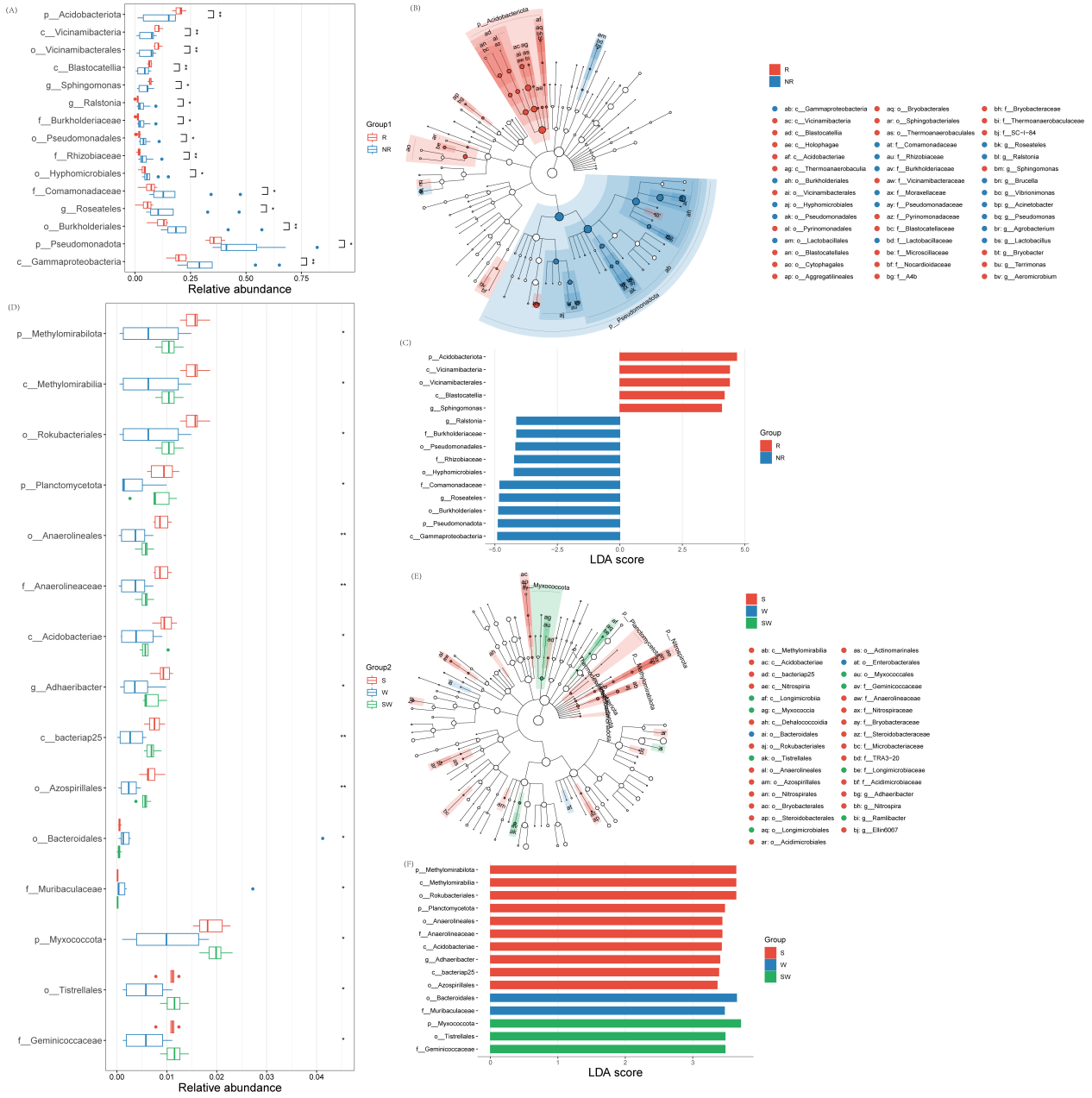


**FIGURE S1** Effect of different soil sampling (bulk soil (NR) and rhizosphere soil (R)) and intercropping systems (S, soybean monoculture; W, wheat monoculture; SW, wheat intercropping with wheat) on relative abundance (A and D), and LeFSe (B, C and E, F) of soil bacterial.


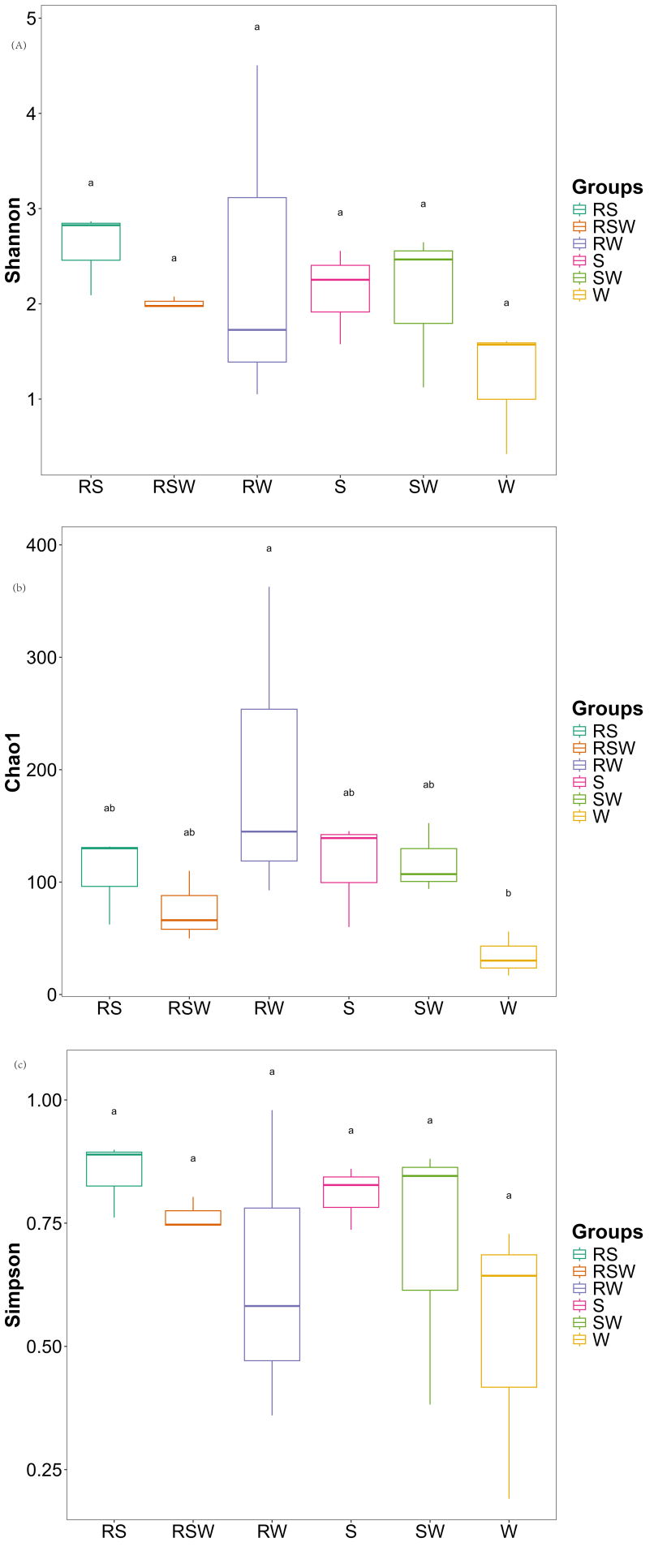


**FIGURE S2** Effect of sampling location and intercropping systems on α-diversity of soil [eukaryote](https://fanyi.so.com/?src=onebox#eukaryote) (ANOVA followed by LSD test; p< 0.05). A represented Shannon diversity index, B represented Chao 1, C represented simpson index.


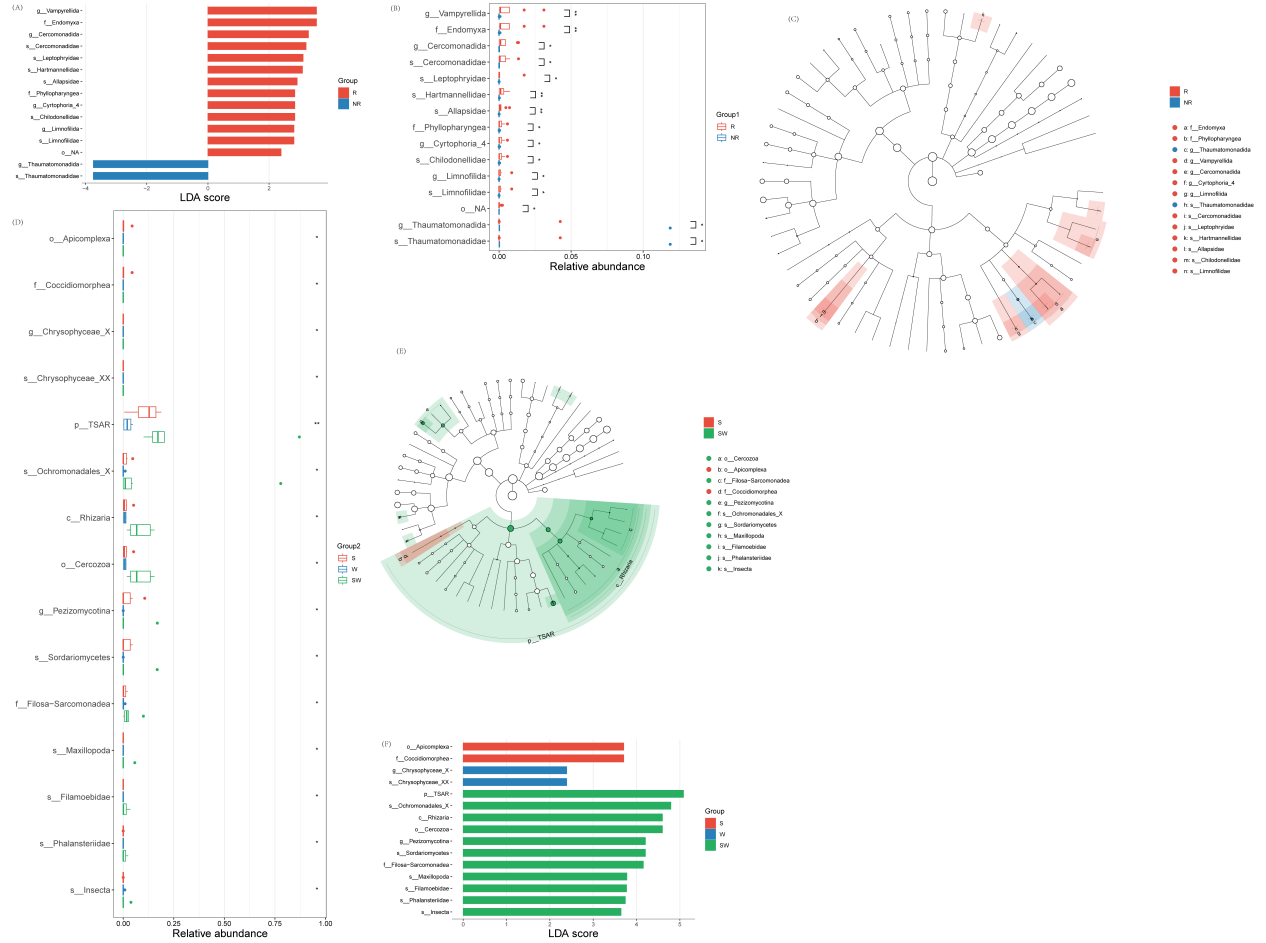


**FIGURE S3** Effect of different soil sampling (bulk soil (NR) and rhizosphere soil (R)) and intercropping systems (S, soybean monoculture; W, wheat monoculture; SW, wheat intercropping with wheat) on relative abundance (B and D), and LeFSe (A, C and E, F) of soil eukaryotic.

**Table A1** Analysis of Variance (ANOVA) of α-diversity of soil bacterial and eukaryotic under different soil sampling and intercropping systems

| Treatments |  | Soil bacterial | | |  | Soil eukaryotic | | |
| --- | --- | --- | --- | --- | --- | --- | --- | --- |
|  |  | Shannon | Chao 1 value | Simpson |  | Shannon | Chao 1 value | Simpson |
| RS |  | 6.80±0.53a | 13832.06±12550.90a | 0.9930±0.003a |  | 2.59±0.44a | 107.98±39.54ab | 0.850±0.077a |
| RW |  | 6.73±0.39a | 10865.01±6352.59a | 0.9935±0.003a |  | 2.43±a1.83a | 200.10±143.21a | 0.640±0.314a |
| RSW |  | 6.61±0.39a | 12858.44±7258.18a | 0.9866±0.009a |  | 2.01±0.06a | 75.37±31.05ab | 0.765±0.033a |
| S |  | 6.59±0.14a | 8126.31±2280.51a | 0.9920±0.001a |  | 2.13±0.50a | 114.86±47.61ab | 0.808±0.064a |
| W |  | 3.74±1.58b | 2208.62±1518.55a | 0.8547±0.107b |  | 1.20±0.67a | 34.40±19.84b | 0.521±0.289a |
| SW |  | 6.31±0.36a | 7993.37±1365.56a | 0.9831±0.010a |  | 2.08±0.83a | 117.87±30.70ab | 0.703±0.278a |
| **Block** |  | 1.44ns | 1.32ns | 0.850ns |  | 0.43ns | 0.017ns | 0.71ns |
| **Sampling location (L)** |  | 1.64ns | 4.5ns | 5.188* |  | 1.44ns | 0.364ns | 0.53ns |
| **Bulk soil** |  | 5.55b | 6109.43a | 0.943b |  | 1.80a | 89.04a | 0.68a |
| **Rhizosphere** |  | 6.71a | 12518.5a | 0.991a |  | 2.34a | 127.82a | 0.75a |
| **Intercropping systems (T)** |  | 0.99ns | 0.86ns | 4.268* |  | 0.5ns | 3.5ns | 1.99ns |
| W |  | 5.24b | 6536.81a | 0.924b |  | 1.81a | 117.25a | 0.58ns |
| SW |  | 6.46a | 10425.90a | 0.985b |  | 2.04a | 96.62a | 0.73a |
| S |  | 6.69a | 10979.19a | 0.992a |  | 2.36a | 111.42a | 0.83a |
| L×T |  | 7.4* | 0.14ns | 4.718* |  | 0.7ns | 3.5ns | 0.05ns |

**Note:** the values represent the mean±SD. Different lowercase letters in the same column show significant difference among intercropping treatments (p < 0.05). “*” and “**” represent significance at 5% and 1% levels, respectively. W, bulk soil in wheat monoculture; SW, wheat intercropped with soybean; S, soybean monoculture; RW, rhizosphere soil in wheat monoculture; RSW, rhizosphere soil in wheat intercropped with soybean; RS, rhizosphere soil in soybean monoculture.

**Table A2** PCoA (Principal Coordinate Analysis) for soil bacterial and eukaryotic communities among different intercropping systems in different soil sampling location (bulk soil and rhizosphere soil) and significance levels according to PERMANOVA for impacts of soil sampling and intercropping practices on soil bacterial and eukaryotic communities.

| **Factor** |  | **Df** |  | **Soil bacterial** | | |  | **Soil eukaryotic** | | |
| --- | --- | --- | --- | --- | --- | --- | --- | --- | --- | --- |
|  |  |  |  | F | R^2^ | *p* |  | F | R^2^ | *p* |
| Soil sampling (A) |  | 1 |  | 3.77 | 0.19 | 0.013 |  | 0.79 | 0.047 | 0.75 |
| Intercropping systems (B) |  | 2 |  | 1.48 | 0.16 | 0.17 |  | 1.05 | 0.12 | 0.38 |
| A×B |  | 5 |  | 2.90 | 0.55 | 0.008 |  | 1.32 | 0.35 | 0.038 |

**Note:** Df, degree of freedom, F, the P-value, and the proportion of variance (R^2^) explained by each factor refer to the total variance reported.

**TABLE A5** Topology of co-occurrence networks and complexity constructed for soil bacterial and eukaryotic communities among intercropping systems (corresponding to Figs. 4 and 5).

| **Network metrics** | **Soil bacterial** | | |  | **Soil eukaryotic** | | |
| --- | --- | --- | --- | --- | --- | --- | --- |
|  | W | SW | S |  | W | SW | S |
| **num.edges (Links)** | **1179** | **2083** | **2179** |  | **182** | **513** | **206** |
| num.pos.edges | 773 | 1064 | 1227 |  | 140 | 408 | 203 |
| num.neg.edges | 406 | 1019 | 952 |  | 42 | 105 | 3 |
| **num.vertices (Nodes)** | **197** | **272** | **282** |  | **85** | **79** | **93** |
| connectance | 0.06 | 0.06 | 0.05 |  | 0.05 | 0.17 | 0.05 |
| average.degree | 11.97 | 15.32 | 15.45 |  | 4.28 | 12.99 | 4.43 |
| average.path.length | 3.64 | 3.22 | 3.24 |  | 4.26 | 2.31 | 2.18 |
| edge.connectivity | 0 | 1 | 0 |  | 0 | 4 | 0 |
| clustering.coefficient | 0.49 | 0.47 | 0.51 |  | 0.37 | 0.65 | 0.61 |
| no.clusters | 2 | 1 | 2 |  | 4 | 1 | 10 |
| centralization.degree | 0.18 | 0.16 | 0.15 |  | 0.09 | 0.14 | 0.10 |
| centralization.betweenness | 0.1 | 0.03 | 0.06 |  | 0.23 | 0.07 | 0.01 |
| centralization.closeness | 1.42 | 0.2 | 1.36 |  | 1.42 | 0.2 | 0.91 |
| diameter | 10 | 10 | 9 |  | 10 | 4 | 7 |
| Complexity | 0.82 | 0.95 | 0.98 |  | 0.38 | 1 | 0.49 |

**Note:** S, soybean monoculture; W, wheat monoculture; SW, wheat intercropping with wheat.

**Table A6** Robustness-bar (random_removal_result) of soil bacterial and eukaryotic

|  | **Soil bacterial** | | | |  | **Soil eukaryotic** | | | | |
| --- | --- | --- | --- | --- | --- | --- | --- | --- | --- | --- |
|  | Proportion.removed | remain.mean | remain.sd | remain.se |  | Proportion.removed | remain.mean | remain.sd | remain.se | group |
| 1 | 0.05 | 0.44893 | 0.01381 | 0.00138 |  | 0.05 | 0.70316 | 0.02314 | 0.00231 | SW |
| 2 | 0.1 | 0.43250 | 0.01703 | 0.00170 |  | 0.1 | 0.65139 | 0.03175 | 0.00317 | SW |
| 3 | 0.15 | 0.40051 | 0.01809 | 0.00181 |  | 0.15 | 0.60582 | 0.04027 | 0.00403 | SW |
| 4 | 0.2 | 0.38493 | 0.01819 | 0.00182 |  | 0.2 | 0.55734 | 0.03984 | 0.00398 | SW |
| 5 | 0.25 | 0.36018 | 0.02053 | 0.00205 |  | 0.25 | 0.51861 | 0.04603 | 0.00460 | SW |
| 6 | 0.3 | 0.33228 | 0.01868 | 0.00187 |  | 0.3 | 0.49089 | 0.04152 | 0.00415 | SW |
| 7 | 0.35 | 0.31007 | 0.02263 | 0.00226 |  | 0.35 | 0.44899 | 0.04175 | 0.00417 | SW |
| 8 | 0.4 | 0.28540 | 0.02181 | 0.00218 |  | 0.4 | 0.39797 | 0.04693 | 0.00469 | SW |
| 9 | 0.45 | 0.25820 | 0.02110 | 0.00211 |  | 0.45 | 0.36671 | 0.04991 | 0.00499 | SW |
| **10** | **0.5** | **0.23838** | **0.02191** | **0.00219** |  | **0.5** | **0.33089** | **0.04200** | **0.00420** | **SW** |
| 11 | 0.55 | 0.20625 | 0.01992 | 0.00199 |  | 0.55 | 0.30266 | 0.04570 | 0.00457 | SW |
| 12 | 0.6 | 0.18320 | 0.01784 | 0.00178 |  | 0.6 | 0.26342 | 0.03989 | 0.00399 | SW |
| 13 | 0.65 | 0.15592 | 0.01978 | 0.00198 |  | 0.65 | 0.23443 | 0.03444 | 0.00344 | SW |
| 14 | 0.7 | 0.12978 | 0.01791 | 0.00179 |  | 0.7 | 0.18924 | 0.03392 | 0.00339 | SW |
| 15 | 0.75 | 0.10746 | 0.01663 | 0.00166 |  | 0.75 | 0.14304 | 0.03082 | 0.00308 | SW |
| 16 | 0.8 | 0.08110 | 0.01455 | 0.00145 |  | 0.8 | 0.10595 | 0.02848 | 0.00285 | SW |
| 17 | 0.85 | 0.05710 | 0.01179 | 0.00118 |  | 0.85 | 0.07038 | 0.02622 | 0.00262 | SW |
| 18 | 0.9 | 0.03154 | 0.01028 | 0.00103 |  | 0.9 | 0.03633 | 0.02179 | 0.00218 | SW |
| 19 | 0.95 | 0.01143 | 0.00762 | 0.00076 |  | 0.95 | 0.00861 | 0.01032 | 0.00103 | SW |
| 20 | 1 | 0.00000 | 0.00000 | 0.00000 |  | 1 | 0.00000 | 0.00000 | 0.00000 | SW |
| 21 | 0.05 | 0.69822 | 0.01105 | 0.00110 |  | 0.05 | 0.58012 | 0.02046 | 0.00205 | W |
| 22 | 0.1 | 0.65553 | 0.01546 | 0.00155 |  | 0.1 | 0.53718 | 0.02056 | 0.00206 | W |
| 23 | 0.15 | 0.61046 | 0.01825 | 0.00182 |  | 0.15 | 0.48482 | 0.02734 | 0.00273 | W |
| 24 | 0.2 | 0.57081 | 0.02021 | 0.00202 |  | 0.2 | 0.45071 | 0.02836 | 0.00284 | W |
| 25 | 0.25 | 0.53239 | 0.02173 | 0.00217 |  | 0.25 | 0.40894 | 0.03382 | 0.00338 | W |
| 26 | 0.3 | 0.48538 | 0.02098 | 0.00210 |  | 0.3 | 0.36788 | 0.03648 | 0.00365 | W |
| 27 | 0.35 | 0.45076 | 0.02061 | 0.00206 |  | 0.35 | 0.32400 | 0.03726 | 0.00373 | W |
| 28 | 0.4 | 0.41234 | 0.02469 | 0.00247 |  | 0.4 | 0.28600 | 0.03149 | 0.00315 | W |
| 29 | 0.45 | 0.36914 | 0.02264 | 0.00226 |  | 0.45 | 0.26271 | 0.03932 | 0.00393 | W |
| **30** | **0.5** | **0.33025** | **0.02447** | **0.00245** |  | **0.5** | **0.21882** | **0.03975** | **0.00397** | **W** |
| 31 | 0.55 | 0.29574 | 0.02178 | 0.00218 |  | 0.55 | 0.18271 | 0.03966 | 0.00397 | W |
| 32 | 0.6 | 0.25122 | 0.02547 | 0.00255 |  | 0.6 | 0.15565 | 0.03539 | 0.00354 | W |
| 33 | 0.65 | 0.21391 | 0.02275 | 0.00227 |  | 0.65 | 0.12988 | 0.03889 | 0.00389 | W |
| 34 | 0.7 | 0.17411 | 0.02067 | 0.00207 |  | 0.7 | 0.09235 | 0.03317 | 0.00332 | W |
| 35 | 0.75 | 0.13685 | 0.02019 | 0.00202 |  | 0.75 | 0.07376 | 0.02530 | 0.00253 | W |
| 36 | 0.8 | 0.10482 | 0.01820 | 0.00182 |  | 0.8 | 0.04400 | 0.02392 | 0.00239 | W |
| 37 | 0.85 | 0.07025 | 0.01860 | 0.00186 |  | 0.85 | 0.02788 | 0.01686 | 0.00169 | W |
| 38 | 0.9 | 0.04000 | 0.01299 | 0.00130 |  | 0.9 | 0.01212 | 0.01423 | 0.00142 | W |
| 39 | 0.95 | 0.01218 | 0.00851 | 0.00085 |  | 0.95 | 0.00271 | 0.00666 | 0.00067 | W |
| 40 | 1 | 0.00000 | 0.00000 | 0.00000 |  | 1 | 0.00000 | 0.00000 | 0.00000 | W |
| 41 | 0.05 | 0.58642 | 0.01204 | 0.00120 |  | 0.05 | 0.63538 | 0.02034 | 0.00203 | S |
| 42 | 0.1 | 0.54833 | 0.01551 | 0.00155 |  | 0.1 | 0.59312 | 0.02637 | 0.00264 | S |
| 43 | 0.15 | 0.51770 | 0.01808 | 0.00181 |  | 0.15 | 0.53785 | 0.03264 | 0.00326 | S |
| 44 | 0.2 | 0.47986 | 0.01864 | 0.00186 |  | 0.2 | 0.49516 | 0.03628 | 0.00363 | S |
| 45 | 0.25 | 0.44805 | 0.02009 | 0.00201 |  | 0.25 | 0.44925 | 0.03689 | 0.00369 | S |
| 46 | 0.3 | 0.40947 | 0.02242 | 0.00224 |  | 0.3 | 0.40570 | 0.03551 | 0.00355 | S |
| 47 | 0.35 | 0.37883 | 0.02183 | 0.00218 |  | 0.35 | 0.35978 | 0.03276 | 0.00328 | S |
| 48 | 0.4 | 0.34333 | 0.02329 | 0.00233 |  | 0.4 | 0.31624 | 0.03992 | 0.00399 | S |
| 49 | 0.45 | 0.30628 | 0.02302 | 0.00230 |  | 0.45 | 0.26753 | 0.04085 | 0.00408 | S |
|  | **0.5** | **0.27918** | **0.01904** | **0.00190** |  | **0.5** | **0.24022** | **0.03754** | **0.00375** | **S** |
| 51 | 0.55 | 0.24922 | 0.02280 | 0.00228 |  | 0.55 | 0.19925 | 0.03698 | 0.00370 | S |
| 52 | 0.6 | 0.21518 | 0.01841 | 0.00184 |  | 0.6 | 0.15570 | 0.03748 | 0.00375 | S |
| 53 | 0.65 | 0.18351 | 0.01920 | 0.00192 |  | 0.65 | 0.12839 | 0.03313 | 0.00331 | S |
| 54 | 0.7 | 0.15351 | 0.01779 | 0.00178 |  | 0.7 | 0.09753 | 0.03229 | 0.00323 | S |
| 55 | 0.75 | 0.11897 | 0.01606 | 0.00161 |  | 0.75 | 0.06591 | 0.02862 | 0.00286 | S |
| 56 | 0.8 | 0.09021 | 0.01653 | 0.00165 |  | 0.8 | 0.05140 | 0.02351 | 0.00235 | S |
| 57 | 0.85 | 0.05961 | 0.01261 | 0.00126 |  | 0.85 | 0.02914 | 0.01971 | 0.00197 | S |
| 58 | 0.9 | 0.03603 | 0.01112 | 0.00111 |  | 0.9 | 0.01129 | 0.01300 | 0.00130 | S |
| 59 | 0.95 | 0.01181 | 0.00718 | 0.00072 |  | 0.95 | 0.00247 | 0.00609 | 0.00061 | S |
| 60 | 1 | 0.00000 | 0.00000 | 0.00000 |  | 1 | 0.00000 | 0.00000 | 0.00000 | S |

**TABLE A7** Result of Vulnerability index

| **Treatments** | **Soil bacterial** |  | **Soil eukaryotic** |
| --- | --- | --- | --- |
| SW | 0.0157134183363691 |  | 0.0216436328082142 |
| W | 0.0192296384737454 |  | 0.169040650910159 |
| S | 0.00838300087239668 |  | 0.046729369669236 |

**Note:** S, soybean monoculture; W, wheat monoculture; SW, wheat intercropping with wheat.

**TABLE A8** Distance-based redundancy analysis (dbRDA) of soil bacterial at ASVs level and soil properties

| **Properties** | **Sum of Sqs** | **F** | **Pr(>F)** |
| --- | --- | --- | --- |
| **TP** | **0.456594386489121** | **6.72230433157863** | **0.003** |
| TN | 0.0967278301668314 | 1.42409528228001 | 0.243 |
| **AP** | **0.211686126201088** | **3.11659233053357** | **0.023** |
| **pH** | **0.271200928356818** | **3.99281118946613** | **0.012** |
| SOC | 0.136485164927237 | 2.00943078262844 | 0.106 |
| Cs | 0.0958007446289639 | 1.41044607565075 | 0.239 |
| Us | 0.0718093255849284 | 1.05722749712081 | 0.417 |
| **Su** | **0.324434474055515** | **4.77655296427694** | **0.003** |
| Pm | 0.0972101631142559 | 1.4311965278443 | 0.234 |
| phosphatase | 0.125547886946757 | 1.84840446842165 | 0.145 |
| **WSOC** | **0.389092621259956** | **5.7284957736628** | **0.003** |
| **EOC** | **0.249839542051577** | **3.67831380636774** | **0.017** |
| SN | 0.0896927293475382 | 1.32051957020377 | 0.264 |
| H_2_O_2_ | 0.110686043757193 | 1.62959793946571 | 0.192 |
| Residual | 0.203766906688928 | NA | NA |

**Note:** TP, The total phosphorus (g kg^-1^); TN, The total nitrogen content (g kg^-1^); AP, soil available phosphorus (mg kg^-1^); SOC, the soil organic carbon content (g kg^-1^); Cs, Soil electrical conductivity (us cm^-1^); WSOC, soil water-soluble organic carbon content (g kg^-1^); EOC, soil easily oxidizable organic matter (mg g^-1^); SN, nitrate nitrogen (mg kg^-1^); Us, The soil urease activity (mg kg^-1^ h^-1^); Su, the soil sucrose activity (mg g^-1^ h^-1^); Pm, the soil microbral phosphorus (mg kg^-1^); H_2_O_2_, the catalase activity (g kg^-1^ 20min^-1^).

**TABLE A9** Distance-based redundancy analysis (dbRDA) of soil eukaryotic at ASVs level and soil properties

| **Properties** | **Sum of Sqs** | **F** | **Pr(>F)** |
| --- | --- | --- | --- |
| TP | 0.299590234095754 | 0.931040011982266 | 0.569 |
| TN | 0.358561907686024 | 1.11430695942405 | 0.311 |
| AP | 0.399033085195777 | 1.24007970267567 | 0.236 |
| pH | 0.344230908626187 | 1.06977034902132 | 0.401 |
| SOC | 0.504285311560107 | 1.567173256 | 0.055 |
| Cs | 0.514596686115341 | 1.59921803276715 | 0.053 |
| Us | 0.307512625706601 | 0.955660519398185 | 0.499 |
| Su | 0.436825038795652 | 1.35752619100551 | 0.135 |
| Pm | 0.418681696243593 | 1.30114191693846 | 0.175 |
| phosphatase | 0.499962931031491 | 1.55374054399074 | 0.074 |
| WSOC | 0.502692000812468 | 1.56222170549872 | 0.059 |
| **EOC** | **0.519086122452224** | **1.61316990564279** | **0.048** |
| SN | 0.481301581080003 | 1.49574645237797 | 0.089 |
| H_2_O_2_ | 0.276596719446268 | 0.859582802372658 | 0.656 |
| Residual | 0.965340576903563 | NA | NA |

**Note:** TP, The total phosphorus (g kg^-1^); TN, The total nitrogen content (g kg^-1^); AP, soil available phosphorus (mg kg^-1^); SOC, the soil organic carbon content (g kg^-1^); Cs, Soil electrical conductivity (us cm^-1^); WSOC, soil water-soluble organic carbon content (g kg^-1^); EOC, soil easily oxidizable organic matter (mg g^-1^); SN, nitrate nitrogen (mg kg^-1^); Us, The soil urease activity (mg kg^-1^ h^-1^); Su, the soil sucrose activity (mg g^-1^ h^-1^); Pm, the soil microbral phosphorus (mg kg^-1^); H_2_O_2_, the catalase activity (g kg^-1^ 20min^-1^).
